# Subperiosteal Aneurysmal Bone Cyst with Florid Ossification: A Rare Subtype

**DOI:** 10.1155/2020/8893963

**Published:** 2020-11-05

**Authors:** Talal Ahmad, Rana Naous

**Affiliations:** SUNY Upstate Medical University, Syracuse, USA

## Abstract

Aneurysmal bone cyst (ABC) is a well-characterized benign cystic lesion of the bone with common localization to the medulla of the long bones. Rarely, ABCs may arise within the subperiosteal region, which can be diagnostically challenging for both the radiologist and pathologist due to their aggressive radiologic appearance thus mimicking other malignant neoplasms. Herein, we present a rare case of subperiosteal ABC with prominent soft tissue involvement and florid reactive periosteal ossification and provide a short literature review on subperiosteal ABCs.

## 1. Introduction

ABCs comprise approximately 1% of all primary bone tumors and tend to occur within the first 2 decades of life [1]. However, any age group may be affected and no sexual predilection has been observed. The rare subperiosteal ABCs have a relative incidence of approximately 1617% compared to classic ABCs and occur in a slightly older age group [2]. Surface ABCs more commonly affect the diaphysis compared to intramedullary classic ABCs that often involve the metaphysis [2, 3]. The most commonly affected sites of classic ABCs are the long bones of the femur and tibia, as well as the vertebral column [4]. On the other hand, subperiosteal ABCs have never been reported so far to affect flat bones or the spine and tend to predominantly involve long bones. Upon presentation, the most common symptoms are pain and swelling with a limited range of motion at the affected site [5]. A palpable mass may also be present. Patients may present with a pathologic fracture near the affected site as the heralding event. If tumors are present in the vertebral column, neurological symptoms involving nerve compression may also be present.

## 2. Case Report

A 36-year-old male with no significant past medical history presented with a 6-8 week history of unresolving pain in his right upper arm that began after lifting a heavy piece of cast iron. Physical examination revealed swelling and tenderness over the midshaft of the right humerus.

X-ray showed a lytic, expansile exophytic surface lesion on the humeral diaphysis with a thin rim of calcification around it. MRI showed a more aggressive lesion, measuring 9 cm, with extension into the surrounding soft tissue and extensive intramedullary fluid signal. A bone scan was also performed which showed intense activity in the proximal humerus.

**Figure 1:**
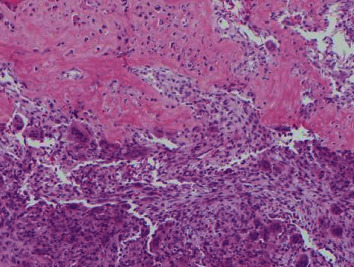
Cellular fibrous septa containing uniform fibroblasts with scattered osteoclast-like giant cells adjacent to an area with prominent reactive woven bone deposition in subperiosteal ABC (H&E, 10x).

**Figure 2:**
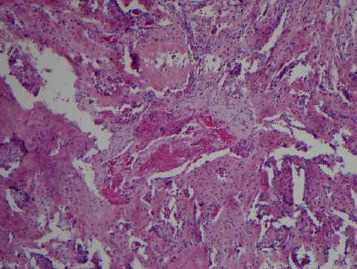
Cyst space in subperiosteal ABC with no lining cells and surrounded by bland fibroblasts and osteoclast type giant cells (H&E, 4x).

**Figure 3:**
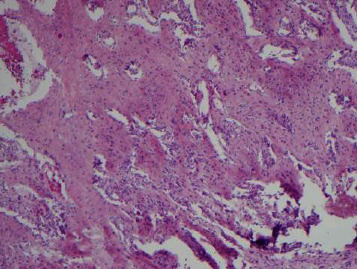
Low-power view demonstrating extensive woven bone formation associated with subperiosteal ABC (H&E, 4x).

**Figure 4:**
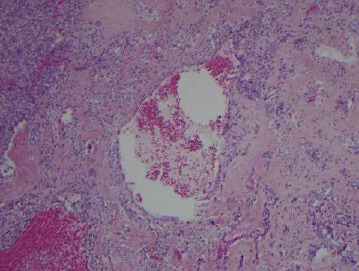
Areas with extensive sheet-like reactive bone deposition in subperiosteal ABC (H&E, 4x).

**Figure 5:**
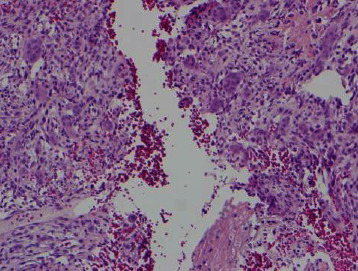
Small cystic hemorrhagic area associated with florid reactive bone formation within the cyst wall in subperiosteal ABC (H&E, 4x).

**Figure 6:**
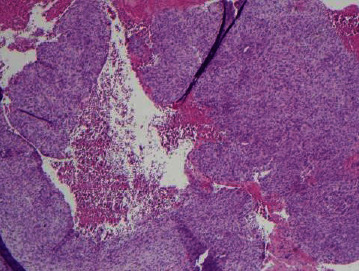
Osteoclast type giant cells in subperiosteal ABC clustering around hemorrhagic spaces (H&E, 10x).

An open biopsy of the surface lesion was then performed with a final diagnosis of “a giant-cell rich lesion with prominent osteoid matrix formation”. Histologically, the diagnosis favored ABC, with a differential that also included subperiosteal hamartoma, periosteal chondroma, and giant cell tumor (GCT). However, the prominent soft tissue involvement as well as the extensive intramedullary signal abnormality on MRI was unusual for a classic ABC, making it difficult to distinguish from periosteal osteosarcoma and telangiectatic osteosarcoma. Therefore, a second open biopsy was then performed with the express intent of thorough removal of the surface bone lesion and open biopsy of the intramedullary lesion. The entire surface lesion, which measured 4.0 × 3.5 × 2.5 cm, was excised and was consistent with subperiosteal ABC. Microscopic examination of the surface lesion showed cellular fibrous septa containing uniform fibroblasts with scattered osteoclast-like giant cells lining variably sized hemorrhagic cystic spaces (Figures 1 and 2). A delicate but prominent meshwork of osteoid and woven bone spicules was seen deposited parallel to the hemorrhagic spaces (Figures 3–5). The osteoclast type giant cells were noted to cluster around the hemorrhagic foci within the septae (Figure 6). The overall findings were most consistent with subperiosteal ABC.

Frozen section evaluation of the intramedullary lesion showed marrow fat and chronic inflammation which likely represented reactive marrow associated with the surface bone lesion.

Intralesional curettage and grafting was then performed with cerement packing, prophylactic stabilization with Synthes large fragment non-locking screws and plate and complex wound closure. On follow-up, the patient reported feeling well with no complaints.

## 3. Discussion

ABC was first described as a distinct entity in a series of case reports written by Jaffe and Lichtenstein in 1942 [6]. It was initially thought to be a reactive process caused by increased venous pressure resulting in dilatation and subsequent rupture of the local vascular network. Only very recently was the clonal neoplastic nature of ABCs uncovered by Nayak et al. [7]. Its primary etiology has been regarded as an arteriovenous fistula within the bone. The lesion may occur in virtually any bone of the body; however, the metaphysis of the long bones is the most common site, though the uncommon “subperiosteal ABCs” have a subperiosteal localization and are not very well characterized in the literature.

The classic ABC usually develops within the medullary cavity of the long bones and causes thinning of the surrounding bone cortex with subsequent protrusion from the bone [8]. Capanna et al. devised a classification scheme of ABCs into five radiological categories [9]. The subperiosteal, type V, ABC tends to be radiologically similar to the classic ABC; however, rather than being an eccentric medullary lesion, it arises as an exophytic mass from the cortical bone with variable extraosseous/soft tissue extension, hence its aggressive appearance, and demonstrates a hyperattenuating rim secondary to the prominent reactive membranous ossification of the periosteum [10]. Scalloping of the underlying cortex is also often seen. Type 1 presentations represent the classical central metaphyseal lesion; well contained within the bone, while type IV presentations represent lesions with subperiosteal extension. Radiographically, type IV lesions consist of a lytic, expansile lesion usually arising eccentrically within or on the bone. The tumor is often well circumscribed with thinly sclerotic margins. Imaging may also show expansion of the surrounding bone with a blown-out or soap bubble appearance [8].

Some investigators believe that radiographic findings are often enough to confirm the diagnosis of ABC. However, due to the confounding nature of the lesion to often mimic and even co-exist with other malignant lesions, many authors believe that open biopsy is necessary to histologically confirm the origin of the lesion. Grossly an ABC, whether classic or subperiosteal, appears as a well circumscribed, spongy mass composed of variably sized blood-filled cystic spaces separated by tan-pink, gritty fibrous septa [11]. Approximately 5% of all ABCs are solid [12]. These solid areas are usually tan-white in appearance and should be thoroughly sampled, as they may represent solid portions of the ABC wall or portions of a primary tumor that developed secondary ABC-like changes.

Histologically, both classic and surface ABCs show cellular fibrous septa containing uniform fibroblasts with scattered osteoclast-like giant cells surrounding aneurysmal spaces. A delicate meshwork of osteoid or woven bone spicules deposited parallel to the vascular space surface is usually present in the fibrous septa lining aneurysmal spaces. However, due to the florid reactive periosteal ossification associated with subperiosteal ABC, it tends to have more prominent reactive woven bone deposition compared to classic ABC. The giant cells seen in ABC are commonly related to vascular spaces or hemorrhagic foci in the septa. These giant cells are not as numerous, large, or evenly spaced as in giant cell tumor of the bone. In rare cases, chondroid foci may be present. ABC shows multiple septa with varying degrees of thickness surrounding the hemorrhagic areas. The cystic spaces show no endothelial lining. The septa are composed of cells with a bland-looking spindle or ovoid-shaped morphology near osteoclast type multinucleated giant cells. Mitotic activity is easily identified in the spindle cell component, but atypical mitoses should not be seen. The stroma of the lesion tends to be fibromyxoid (or “loose”), and inflammatory cells are common. This overall appearance is somewhat similar to granulation or repair tissue which led to the traditional or historical belief that ABC was nonneoplastic and reactive in nature [4, 6, 7].

There are no specific immunohistochemical stains that are characteristic for ABC. However, P63 can stain some spindled fibroblasts, which also tend to express smooth muscle actin. Osteoclasts are usually positive for CD68 immunostain.

At the molecular level, Panoutsakopoulos et al. were first to report, in the late 1990s, 2 examples of ABCs that were characterized by the chromosomal translocation t(16;17)(q22;p13). This was the first convincing evidence that supported the notion that ABC was clonal in nature [13]. This contradicted the more popular hypothesis that ABC was the result of a disturbance in the local vasculature that lead to bone destruction and expansion secondary to increased vasculature pressure [14]. The translocation results in the fusion of the promoter region of osteoblast cadherin 11 gene (CDH11) on chromosome 16q22 to the entire coding sequence of the ubiquitin protease USP6 gene [15]. USP6 was found to induce matrix metalloproteinase (MMP) production via activation of nuclear factor *κ*B [16]. The secretion of MMP would then lead to osteolysis, inflammation, and extensive vascularization. Thus, giving ABC its characteristic hemorrhagic and repair-like appearance.

Radiographically, subperiosteal ABC can be difficult to distinguish from periosteal osteosarcomas and telangiectatic osteosarcoma [17, 18]. This is due to the peculiar location of surface ABCs. In addition, subperiosteal ABCs occasionally demonstrate an aggressive radiographic appearance despite their benign nature due to their frequent association with a prominent soft tissue component and/or periosteal reaction. Additionally, other differential diagnoses of subperiosteal ABCs include giant cell tumor (GCT) which can be excluded based on location of the lesion, the size and abundance of the osteoclast type giant cells, and immunostaining for H3F3A (Histone 3.3) G34W immunostain characteristic of GCTs. Subperiosteal hamartoma and periosteal chondroma are also in the differential diagnosis of subperiosteal ABC; however, the presence of cellular fibrous septae with osteoclast type giant cells along with the relative absence of chondroid material argues against such entities [19].

Upon histological examination, one of the keys in assuring an accurate diagnosis is by thorough sampling of the specimen. The prominent woven bone formation in subperiosteal ABCs may be mistaken for periosteal or telangiectatic osteosarcomas histologically; however, the absence of malignant osteoid formation characteristic of osteosarcoma and the bland nature of the spindled fibroblasts in subperiosteal ABC are key distinguishing features that set them apart [9]. Additionally, although most secondary ABCs are most commonly associated with benign neoplasms, in a few cases, the underlying lesion will be malignant, with osteosarcoma being the most common [11]. Therefore, the pathologist must carefully examine all areas of a suspected lesion.

Subperiosteal ABCs can be aggressive lesions with a potential for rapid growth and subsequent pathologic fracture. Generally, they are treated surgically by aggressive curettage or en bloc resection for very large destructive tumors [20]. When the lesion is encountered in an anatomical location that is difficult to access, other methods of treatment can be utilized such as selective arterial embolization [21]. Other treatment modalities also include radiotherapy and intralesional injection with methylprednisolone or calcitonin [22]. Rarely, an ABC may be encountered in an asymptomatic patient where there is a clinically insignificant destruction of the bone. In these cases, the lesion can be closely monitored instead.

Long-term follow-up is crucial for both classic and subperiosteal ABCs in order to monitor for recurrences and any postoperative skeletal deformities. Patients should be monitored on a regular basis for at least 5 years. Recurrence rates usually vary from 20 to 70% depending on the treatment modality used [11]. Most recurrences were found to happen within the first year of surgery [23]. Any patients who received radiation should also be monitored for life due to the rare possibility of developing secondary sarcomas.

## 4. Conclusion

We present a rare case of subperiosteal ABC with prominent soft tissue involvement and florid reactive periosteal ossification. Subperiosteal ABCs are uncommon lesions that may radiologically mimic other malignant neoplasms such as periosteal osteosarcoma and telangiectatic osteosarcoma, due to their aggressive radiographic appearance and membranous periosteal ossification. Therefore, the astute pathologist must be keenly aware of subperiosteal ABCs during intraoperative frozen section evaluation of aggressive subperiosteal bone lesions and should judiciously sample such lesions during gross examinations in order to avoid diagnostic pitfalls.
